# Viral Recombination: Ecology, Evolution, and Pathogenesis

**DOI:** 10.3390/v10070358

**Published:** 2018-07-06

**Authors:** Kenneth M. Stedman

**Affiliations:** Center for Life in Extreme Environments, Department of Biology, Portland State University, 1719 SW 10th Avenue—P.O. Box 751, Portland, OR 97207-0751, USA; kstedman@pdx.edu

Recombination between and within virus genomes is being increasingly recognized as a major driver of virus evolution. Virus evolution can lead to changes in virus pathogenesis and virus ecology. The ubiquity of high-throughput sequencing of multiple virus isolates has revolutionized data acquisition, thus the editors of *Viruses* decided that a Special Issue on viral recombination was appropriate.

This Special Issue contains papers on a variety of viruses, both from the host and genome perspectives. There are manuscripts describing five ssRNA viruses, four ssDNA viruses, and one dsDNA virus, all of which undergo or have undergone varying amounts of recombination. Manuscripts in this Special Issue discuss recombination in two plant viruses and six animal viruses, as well as recombination in a group of viruses discovered by metagenomic analysis, and one virus with an unknown host that is apparently derived from a recombination between DNA and RNA viruses. Two manuscripts describe recombination in the RNA foot and mouth disease viruses (FMDV) in wildlife, Ferretti et al. discuss FMDV in a very narrow host range where recombination seems to dominate [1], and Lasecka-Dykes et al. analyze FMDV in multiple wildlife species in Southern Africa where much less recombination is observed [2]. An analysis of the genomes of vaccine-derived polio strains from the Czech Republic by Korotkova et al. reveals that there are recombination hotspots in these strains, indicating potential targets for vaccine development [3]. Some of these lessons from polio may be applicable to the emerging hand, foot, and mouth disease virus EV-A71, extensively reviewed by Mandary and Poh [4]. Mechanisms of recombination in many viruses are not well understood; however, Ruiz et al., using the model plant RNA virus Brome Mosaic Virus describe data that indicate that the subcellular location of viral RNAs is critical to determine whether they recombine or not [5].

Recombination is not limited to RNA viruses; both ds and ssDNA viruses are known to undergo recombination. A novel set of fluorescent HSV-1 viruses is presented by Law et al. [6] and is used to demonstrate that HSV-1 undergoes recombination during the infection process. A pair of studies on novel extant ssDNA viruses, both those that infect plants (by Fontenele et al. [7]) and animals (by Stenzel et al. [8]), indicate that there is extensive recombination in these genomes. Two extreme examples of recombination in ssDNA viruses are presented by Kazlauskas et al. and Bistolas et al. The first is an analysis of metagenomic sequences in which a large number of so-called CRESS (Circular Rep-Encoding Single Stranded) DNA viruses not only have highly diverse genomes but the Rep protein, responsible for initiating replication of the single stranded DNA, also seems to have undergone rampant recombination between its different domains [9]. Possibly the most extreme example of recombination in this Special Issue is that of a so-called “Crucivirus”, a lineage that appears to have arisen from the recombination between a DNA and RNA virus. This example provides insight into the hosts for this novel group of viruses that may be infecting protists associated with marine crustaceans [10].

I hope that this collection of diverse manuscripts describing recombination in a variety of viruses will lead to a better understanding of virus genome diversity generated by recombination. Clearly there are many aspects of virus recombination that are not included herein. These papers provide an indication of the widespread nature of recombination in virus genomes. The next steps are to understand the mechanism of the recombination processes and determine the role of recombination in pathogenesis and virus evolution.
